# Bioinformatics-based analysis of the association between the A1-chimaerin (*CHN1*) gene and gastric cancer

**DOI:** 10.1080/21655979.2021.1940621

**Published:** 2021-06-21

**Authors:** Jie-Pin Li, Shu-Hong Zeng, Yong-Hua Zhang, Yuan-Jie Liu

**Affiliations:** a Department of Oncology, Zhangjiagang TCM Hospital Affiliated to Nanjing, University of Chinese Medicine, Zhangjiagang, Jiangsu, China; b No. 1 Clinical Medical College, Nanjing University of Chinese Medicine, Nanjing, Jiangsu, China; c Jiangsu Province Hospital of Chinese Medicine, Affiliated Hospital of Nanjing University of Chinese Medicine, Nanjing, Jiangsu, China

**Keywords:** Gastric cancer, CHN1, biomarker, immune cell infiltration, methylation

## Abstract

Gastric cancer (GC) is one of the most common causes of cancer-related deaths worldwide and the identification of additional therapeutic targets and biomarkers has become vital. The A1-chimaerin (*CHN1*) gene encodes a ras-related protein that can be activated or inactivated by binding to GTP or GDP. The present study aimed to assess the expression of *CHN1* in GC tissue and cells, to explore its relationship with GC progression, and to discover the potential mechanisms underlying these associations. The ONCOMINE database and The Cancer Genome Atlas (TCGA) were used to determine the transcriptional levels of *CHN1* in GC. Western blot and immunohistochemistry were used for detecting protein expression. Correlations between *CHN1* levels and the clinical outcomes of GC patients were examined using Kaplan–Meier and Cox regression analyses. Moreover, the CIBERSORT algorithm was used to estimate immune cell infiltration. In GC patients, *CHN1* transcription and CHN1 protein expression were upregulated, and a high expression of *CHN1* was remarkably linked to poor survival in GC patients. *CHN1* expression was associated with immune infiltrates and this gene showed potential involvement in multiple cancer-related pathways. Furthermore, the expression of *CHN1* was correlated with the immunotherapeutic response. Finally, our results indicated that the pro-carcinogenic role of *CHN1* may involve DNA methylation. To our knowledge, this is the first report characterizing *CHN1* expression in GC. Our results show that high *CHN1* levels could be used as a clinical biomarker for poor prognosis and that *CHN1* inhibitors may have potential as anti-cancer drugs.

## Introduction

Gastric cancer (GC) is the third leading cause for cancer mortality worldwide [1–4]. A few cases of GC can be cured surgically, but most patients present with more advanced, inoperable GC. To date, the effective interventions for advanced recurrent and refractory GC have been limited to systemic chemotherapy, radiotherapy, targeted therapy, and immunotherapy, and a sustained response is only observed in few cases. The 5-year overall survival in cases of advanced GC remains between 20% and 30% owing to a lack of known sensitive and specific biomarkers [5]. Thus, there is a need for improving GC stratification and identifying new prognostic factors.

*CHN1*, also known as N-chimaerin, *ARHGAP2*, and *RHOGAP2*, is located in the q31-32.1 region of human chromosome 2. This gene encodes a GTP enzyme-activating protein (Rho GTPase-activating protein, Rho Gap) – a ras-related protein that can be activated or inactivated by binding to GTP or GDP [6]. Rho GTPase and its related signaling complexes interact with other proteins and play a role in the tumor microenvironment, tumor invasion, and metastasis [7,8]. Currently, most research on *CHN1* has been conducted in the field of neurobiology [9], as this gene is expressed in the hippocampus and cerebral cortex of the human brain, where it plays an important role in neural signal transduction, brain development, and synaptic genesis [10]. However, there are few reports regarding the role of *CHN1* in tumorigenesis, especially in GC.

*CHN1* expression was found to significantly differ between GC tissue and normal gastric tissue in our previous systematic analysis of genes differentially expressed in GC. Moreover, the upregulation of *CHN1* expression was found to be associated with poor prognosis in GC patients. Hence, we hypothesized that this gene might be related to GC progression and could be a potential predictive biomarker for GC prognosis. In the present study, we aimed to explore the value of *CHN1* in assessing GC prognosis along with the potential biological functions of the gene, in order to guide subsequent experimental validation and targeted drug development for GC.

## Materials and methods

### Expression analysis

The transcriptional expression of *CHN1* in tumor tissue and normal tissue was first evaluated using the ONCOMINE and TIMER databases [11,12]. The GSE118916 [13], GSE13861 [14], and GSE292972 [15] datasets from the GEO database were used to plot receiver operating characteristic (ROC) curves to further assess the value of *CHN1* expression in discriminating tumors from normal tissue [16].

We collected 15 cases sets of surgically removed GC tissue and paired paracancerous tissue samples from Jiangsu Provincial Hospital of Traditional Chinese Medicine, and we also collected detailed clinical information for all patients. The study protocol was approved by the Ethics Committee of the Affiliated Hospital of Nanjing University of Chinese Medicine, and clinicians and patients provided informed consent for using the tissue for research purposes.

Immunohistochemistry (IHC) was performed using 5-μm sections of paraffin-embedded tissue. The slides were dewaxed and incubated in 6% hydrogen peroxide in methanol to inactivate the endogenous peroxidase, and then, antigen recovery was performed using 0.1% pepsin [17]. After blocking the sections with protein blocking solution, the slides were incubated with an anti-CHN1 polyclonal antibody (Proteintech, China) and immunohistochemical staining was performed according to manufacturer instructions. All slides used for comparison were also processed simultaneously and incubated for development for the same duration. Each section was stained with 3,3′-diaminobenzidene and counterstained with hematoxylin. The differential expression of CHN1 between cancerous and paracancerous tissue was evaluated at 200× magnification (Nikon ECLIPSE Ni-U, Japan). IHC results (intensity and extent of staining) were scored independently by two observers. The staining intensity was graded as follows: 0, negative staining; 1, weak staining; 2, moderate staining; and 3, strong staining. Based on the proportion of positively stained cells per specimen, the extent of staining was graded as follows: 0, no positively stained cells; 1 < 10% positively stained cells; 2, 10–50% positively stained cells; and 3, >50% positively stained cells. The histochemistry score (H-SCORE), which reflects both the ratio of positive cells and the intensity of expression, was calculated as follows:

H-SCORE = ∑ (PI × I) = (percentage of cells with weak intensity of staining × 1) + (percentage of cells with moderate intensity of staining × 2) + (percentage of cells with strong intensity of staining × 3) [18]. In the formula, PI represents the percentage of positive cells in a particular field and I represent the intensity of staining. The range of H-SCOREs can be from 0 to 300, with a higher score representing stronger positive expression.

Western blotting was performed as previously described [19]. Radio immunoprecipitation assay (RIPA) buffer (Beyotime Biotechnology, China) was used to isolate proteins, and then quantification was performed via the Bradford method. Samples containing the same amount of protein (20 µg) were separated using 10% sodium dodecyl sulfate-polyacrylamide gel electrophoresis (SDS-PAGE). Subsequently, the proteins were transfer-embedded onto polyvinylidene fluoride (PVDF) membranes. Thereafter, the membranes were blocked with 5% bovine serum albumin and then incubated with a primary anti-CHN1 polyclonal antibody (1:1,000; Proteintech, China). Both the primary antibody and secondary antibody (1: 5,000) were purchased from Zhongshan Golden Bridge Biotechnology (Beijing, China) and added for the binding reaction. After washing, the samples were incubated at 4°C for 1.5 h. Exposure was detected using a gel image processing system (ChemiDoc XRS+,USA) to analyze the target/β-actin bands, and the relative amounts of proteins were calculated. All experiments were performed at least thrice.

For both IHC and western blotting, data are reported as means ± standard deviation. Data were analyzed using SPSS 26.0 (SPSS Inc, USA) and presented using GraphPad Prism 8.0 (GraphPad Software, Inc., USA). * P < 0.05, **P < 0.01, ***P < 0.001, and **** P < 0.0001 were considered statistically significant.

### Survival analysis

Kaplan–Meier (KM) curves were plotted based on information from the KMPLOT and GEPIA databases, and we further evaluated the clinical significance of *CHN1* expression levels using TCGA-STAD data [20].

To establish a Cox model for *CHN1* and analyze the clinical value of its expression, raw counts of RNA-sequencing data and corresponding clinical information on *CHN1* were obtained from The Cancer Genome Atlas (TCGA) (https://portal.gdc.cancer.gov/) in January 2020 [14]. Multivariate Cox regression analysis was used to identify independent prognostic factors for *CHN1* expression. A Forest map was used to display P-values and hazard ratios (HRs) with 95% confidence intervals (CIs) for each variable using the ‘forestplot’ R package.

KM survival analysis and log-rank tests were used to compare survival differences between high-*CHN1* vs. low-*CHN1* groups. Temporal ROC analysis was used to compare the prediction accuracy of *CHN1* expression. For the KM curve, P-values and HRs with 95% CIs were generated using the log-rank test and univariate Cox proportional hazards regression.

### Enrichment analysis

First, we imported the *CHN1* gene into the GeneMANIA database to identify its associated genes [21]. TCGA-STAD samples were assigned to high- or low-*CHN1* expression groups in accordance with the median of the *CHN1* RNA expression data. A total of 490 genes whose expression was positively correlated with that of *CHN1* were obtained. A protein–protein interaction network (PPI) including *CHN1* and its positively correlated genes was plotted using the GeNets database [22]. Subsequently, these genes were imported into the Metascape database for enrichment analysis [23] Biological Process (BP), Molecular Function (MF), Cellular Component (CC), and Kyoto Encyclopedia of Genes and Genomes (KEGG) pathway analysis.

Gene Set Enrichment Analysis (GSEA) generated an ordered list of all genes based on their relationship with *CHN1* expression. Following this, predefined gene sets (biological signatures of gene expression under the perturbation of certain cancer-associated genes) were generated to obtain an enrichment score, which was a measure of the statistical evidence for rejecting the null hypothesis that the genes were randomly distributed in an ordered list [24]. The expression level of *CHN1* was used as a phenotype marker, and the ‘Measure of gene sequencing’ was set to Pearson correlation. All other basic and advanced fields were set to default values. Enrichment analysis was performed using the Hallmark gene set data resource (c2. KEGG.v4.0) in the Molecular Signatures Database-MsigDB (http://www.broad. mit.edu/gsea/msigdb/index.jsp) [25]. A normal P-value < 0.05 was considered statistically significant.

### Immunological analysis

To make reliable immune infiltration estimations, we utilized CIBERSORT, an R package tool that can evaluate multiple cell types [26]. All the results from the previously mentioned analysis methods and the R package tool were assessed using the ‘ggplot2ʹ and ‘pheatmap’ packages. In addition, the immune module of the TIMER database was used to further calculate the Spearman coefficient of correlation between *CHN1* expression and that of common immune checkpoints.

### DNA methylation analysis

We evaluated whether the expression of the *CHN1* gene was closely related to *CHN1* gene methylation. The analysis of *CHN1* DNA methylation and the correlation between disease prognosis and *CHN1* methylation values was performed using the Methsurv and MEXPRESS databases [27]. We also downloaded *CHN1* DNA methylation data, transcriptome data, and clinical data from the TCGA database and visualized it using R software to identify the significance of DNA methylation in the promoter region of *CHN1*.

## Results

In the present study, the differential expression of *CHN1* was verified in several GC cell lines and tissues using western blot and IHC. Further, we found a relationship between *CHN1* expression and clinical parameters and prognosis, indicating that *CHN1* expression is an independent predictor of patient prognosis. Enrichment analysis showed that high expression of *CHN1* may contribute to GC progression via multiple pathways. Further, *CHN1* expression was correlated with the abundance of multiple immune cell infiltrates and positively correlated with the expression levels of common immune checkpoint proteins, suggesting that it may influence the effectiveness of immunotherapy against GC. Taken together, our results indicated that CHN1 expression influences tumorigenesis and progression in GC and could therefore be used as a potential therapeutic target and prognosticator.

### Expression of CHN1 in GC

**Figure 1.**
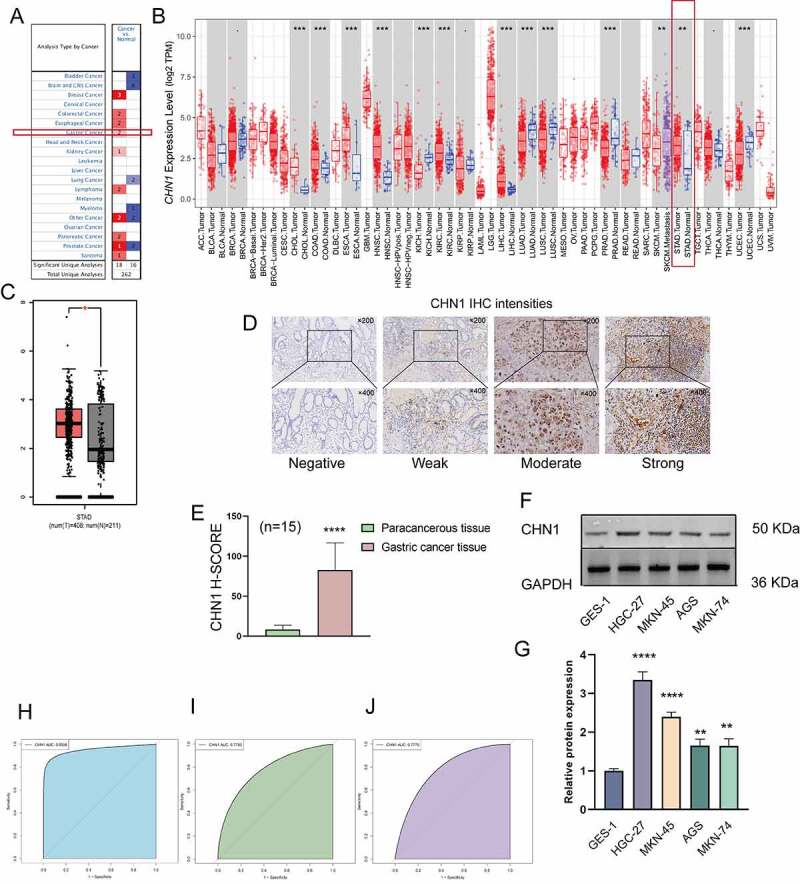
Expression of *CHN1* in gastric cancer (GC). (a-b) Differences in *CHN1* expression between different types of human cancers. (c) Relative *CHN1* expression in GC tissue based on TCGA data (*P < 0.05). (d) Representative images of different immunohistochemical staining intensities for CHN1. (e) Statistical comparison of CHN1 expression levels (H‑SCORE) in paracancerous and GC tissue (n = 15) (****P < 0.0001). (f-g) Differential expression of CHN1 in normal gastric epithelial cells and GC cells (**P < 0.01, ****P < 0.0001). (h-j) Receiver operating characteristic (ROC) curves and areas under the ROC curves for CHN1 expression obtained using three datasets

In order to assess the distinct prognostic and prospective therapeutic significance of differential *CHN1* levels in GC patients, mRNA expression was analyzed using the ONCOMINE data repository (www.oncomine. org) and TIMER (http://timer.cistrome.org/). As indicated in Figure 1(a), the expression of *CHN1* in 20 types of cancers was determined and compared with that in healthy tissues using the ONCOMINE web resource. Furthermore, we also checked for the expression of *CHN1* expression in different types of tumors from the TCGA database via the TIMER web-tool (*P < 0.05, **P < 0.01, ***P < 0.001, and ****P < 0.001). As shown in Figure 1(b), *CHN1* was highly expressed in GC. The GEPIA database also showed that the *CHN1* expression in GC tissue was significantly elevated (Figure 1(c)). Using the IHC analysis of CHN1 expression in 15 sets of GC tumor tissue and paired paracancerous tissue samples, we found that CHN1 immunostaining was cytoplasmic, and the different intensities of IHC staining are shown in Figure 1(d). The mean H-SCORE for CHN1 expression in GC tissues was 83.05, and that in paracancerous tissue was 8.43. Therefore, the expression of CHN1 in tumor tissues was significantly higher than that in paracancerous tissue (P < 0.0001; Figure 1(e)).

Next, we measured the expression of CHN1 in normal gastric epithelial cells and GC cells at different degrees of differentiation using western blot. As shown in figure 1(f-g), the levels of CHN1 in HGC-27 (undifferentiated GC cells), AGS (moderately differentiated GC cells), MKN-45 (poorly differentiated GC cells), and MKN-74 (well-differentiated GC cells) were remarkably different from those in GES-1 (healthy gastric epithelial cells) (**P < 0.01, ****P < 0.0001). An ROC curve was employed to detect the accuracy of CHN1 in distinguishing GC from healthy tissues. The area under the curve was 0.9508 (Figure 1(h)) for GSE118919, 0.7785 (Figure 1(i)) for GSE13861, and 0.7775 (Figure 1(j)) for GSE29272.

### Clinical significance of CHN1 expression in GC

**Figure 2.**
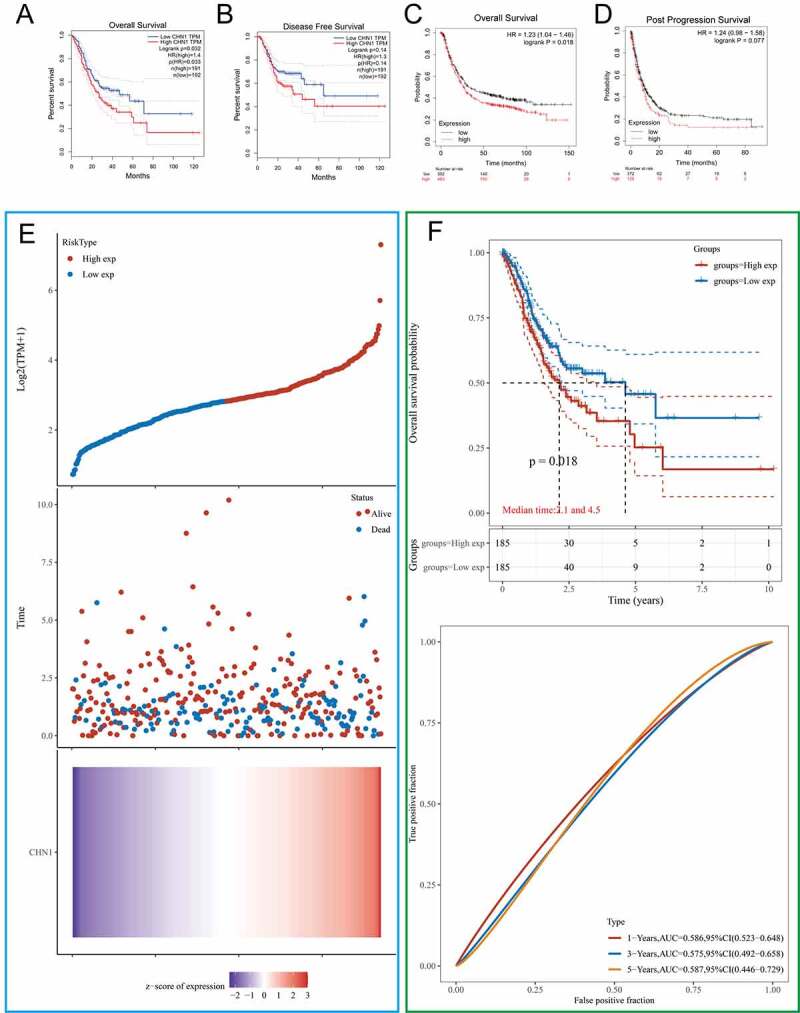
Kaplan–Meier curves comparing survival between patients with high vs. low expression of *CHN1* from three probe sets in the Kaplan-Meier plotter databases and GEPIA. (a) Overall survival (OS) from the GEPIA database. (b) Disease-free survival (DFS) from the GEPIA database. (c) OS from the Kaplan–Meier plotter database. (d) Post-progression survival (PPS) from the Kaplan–Meier plotter database. (e) Curves of risk score. Patients were divided into low-risk and high-risk groups based on median *CHN1* expression. The relationship between survival status and survival duration (years) is illustrated. The horizontal coordinates represent samples, and the samples are ordered consistently. (f) Kaplan–Meier survival analysis and time-dependent receiver operating characteristic curve analysis for *CHN1* gene expression

We first used the GEPIA and KMPLOT databases to clarify the association of *CHN1* expression with overall survival, disease-free survival, and post-progression survival in GC patients. The results revealed that the probability of survival among GC patients with high *CHN1* expression was significantly lower than that among those with low *CHN1* expression (P < 0.05) (Figure 2(a-d)). According to TGCA-STAD data, the survival duration of the low-risk group was significantly longer than that of the high-risk group (Figure 2(e); P < 0.05). Moreover, based on TCGA-STAD data, *CHN1* expression was used to predict the OS of GC patients. The areas under the ROC curves of *CHN1* expression for 1-, 3-, and 5-year survival were 0.586, 0.575, and 0.587, respectively (figure 2(f)).

**Figure 3.**
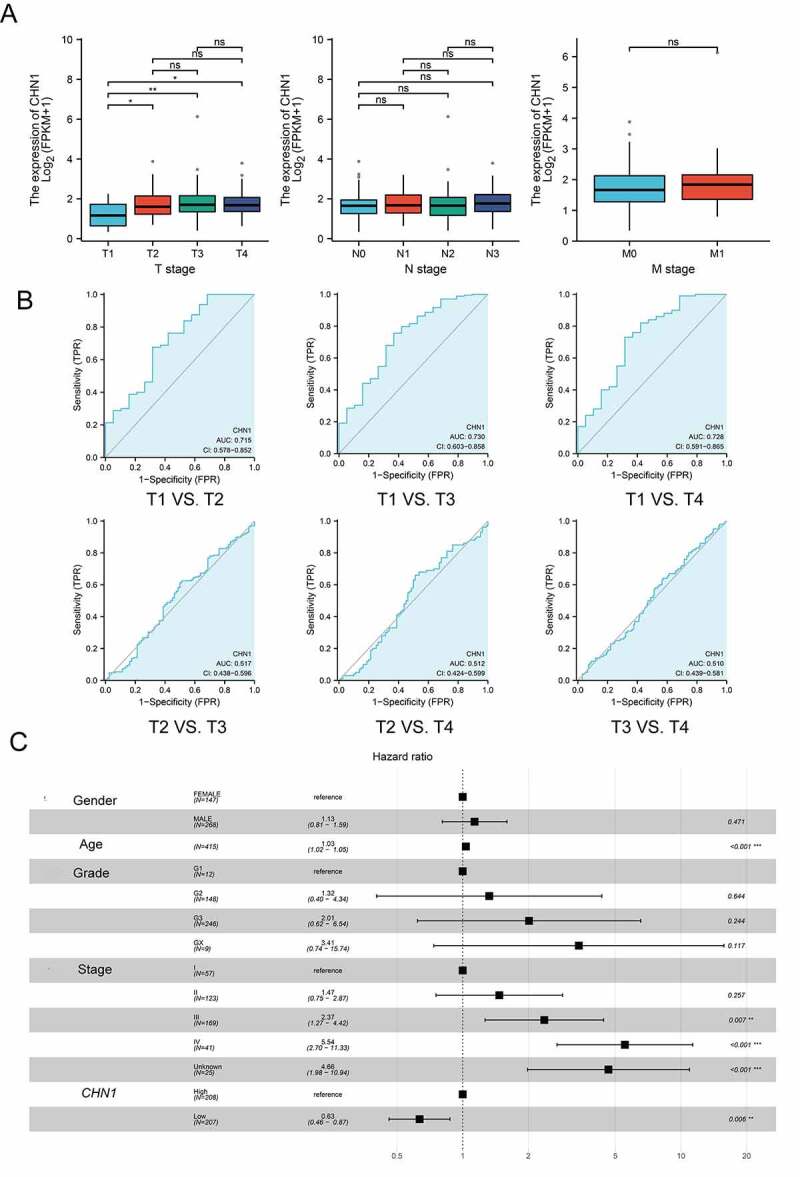
Diagnostic value of *CHN1* levels in GC further analyzed based on clinical characteristics. (a) *CHN1* mRNA expression and its relationship with T, N, and M stages in GC. (b) Receiver operating characteristic curves for T stages. (c) Forest plot constructed by combining information on sex, age, tumor grade, stage, and the expression of *CHN1*. Hazard ratios and P-values of constituents identified using multivariate Cox regression and some parameters related to *CHN1* genes are shown

We then explored the relationship between *CHN1* mRNA expression and T, N, and M stages in GC. The mRNA expression of *CHN1* was distinctly lower at the T1 stage than at other T stages in GC (P < 0.05). However, the mRNA expression of *CHN1* did not show significant differences according to N and M stages (Figure 3(a)).

Therefore, we conducted a subsequent analysis of the diagnostic performance of *CHN1* in differentiating between T stages. The areas of the ROC curves for ‘T1 vs. T2ʹ, ‘T1 vs. T3ʹ, ‘T1 vs. T4ʹ, ‘T2 vs. T3ʹ, ‘T2 vs. T4ʹ, and ‘T3 vs. T4ʹ were 0.715, 0.730, 0.728, 0.517, 0.512, and 0.510, respectively (Figure 3(b)).

Then, a Cox regression model based on TCGA-STAD was used to calculate HRs for different variables. Multivariate analysis revealed that *CHN1* overexpression, age, and tumor stage were all closely corelated with poor prognosis in GC (P < 0.05) (Figure 3(c)). Importantly, *CHN1* expression was verified to be an independent predictor of survival among GC patients.

### Functional enrichment analysis for CHN1

**Figure 4.**
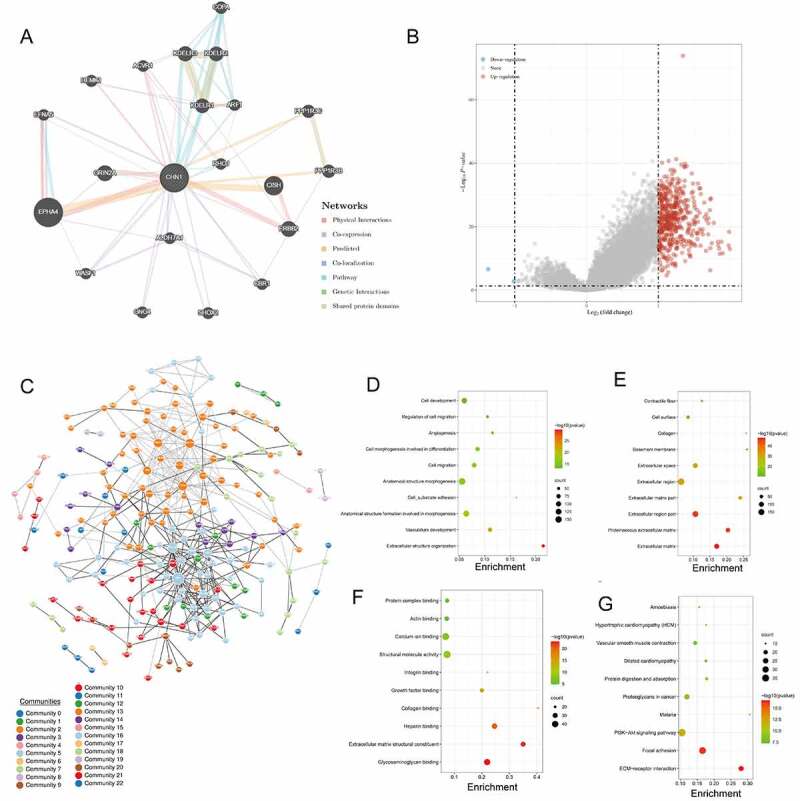
Gene network and enrichment analysis. (a) *CHN1* with its neighboring genes, along with details about its physical interactions, co-expressing genes, predicted, co-localization, functional pathways, genetic interaction, and shared protein domains. (b) Volcano map of differentially expressed genes (DEGs) in response to *CHN1* expression. Red dots represent upregulated genes, and blue dots represent downregulated genes. The abscissa indicates variations in gene expression between different samples (log2 fold change), and the ordinate indicates the significance of the differences (−log10 padj). (c) Network of *CHN1* and the genes that show a positive correlation with its expression, based on the GeNets database. (d) GO: BP, (e) GO: CC, (f) GO: MF (g) KEGG. The color of the circle indicates the adjusted P-value, and the size represents the number of genes enriched in the term or pathway

**Figure 5.**
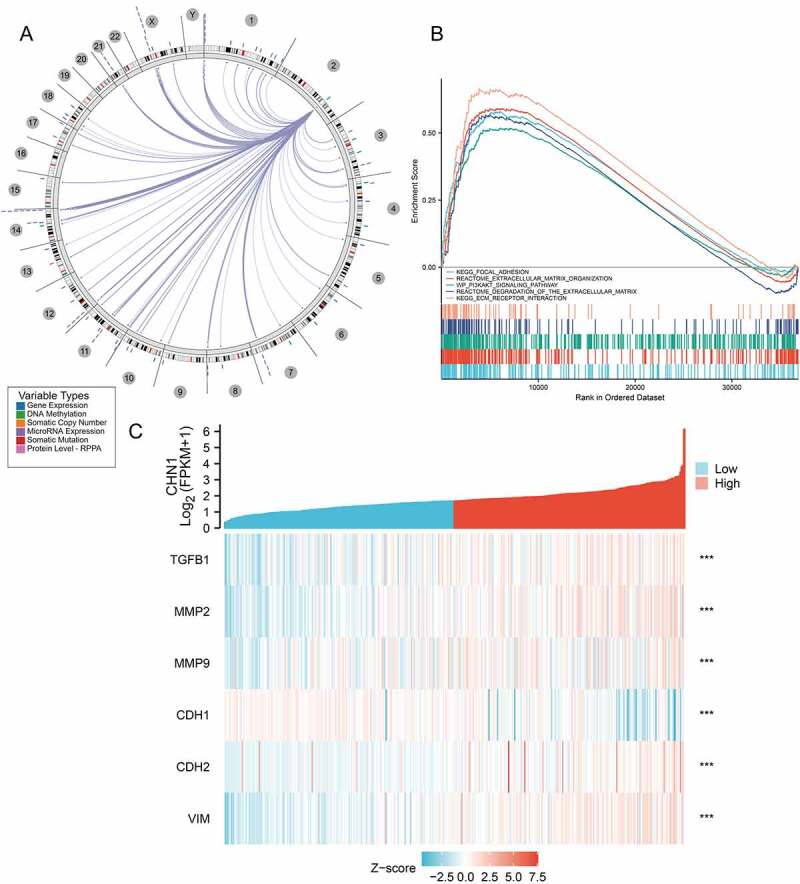
Functional enrichment analysis of *CHN1*.(a) Interrelation between *CHN1* and other genes in GC. (b) GSEA for *CHN1*.(c) Relationship between *CHN1* and six EMT-related factors. Red lines represent a positive correlation and blue lines represent a negative correlation

The constructed functional network was based on the gene function predictions for *CHN1* and was performed using GeneMANIA (Figure 4(a)). The differentially expressed genes (DEGs) identified from TCGA-STAD are presented in Figure 4(b); of these, the genes whose expression was positively correlated with that of *CHN1* were selected to create a PPI network (Figure 5(c)).

Then, the functions of *CHN1* and its related genes were predicted using GO and KEGG analysis to identify the top 10 GO terms and KEGG pathways. Under the BP module, the DEGs were mainly involved in ‘Cell development’, ‘Regulation of cell migration’, ‘Angiogenesis’, and ‘Cell morphogenesis involved in differentiation’ (Figure 4(d)). Under the CC module, the DEGs were mainly concentrated under the terms ‘Contractile fiber’, ‘Cell surface’, ‘Collagen’, ‘Basement membrane’, and ‘Extracellular space’ (Figure 4(e)). In the MF module, the DEGs were primarily enriched under ‘Protein complex binding’, ‘Actin binding’, ‘Calcium ion binding’, ‘Structural molecule activity’, and ‘Integrin binding’ (figure 4(f)). KEGG pathway analysis indicated that the DEGs were mainly involved in ‘Amoebiasis’, ‘Hypertrophic cardiomyopathy (HCM)’, ‘Vascular smooth muscle contraction’, ‘Dilated cardiomyopathy’, and ‘Protein digestion and absorption’ (Figure 4(g)).

Using the Regulome Explorer, we further analyzed the location of the relevant human genes and the correlation between *CHN1* and certain other genes in GC. According to gene associations, DNA methylation, somatic copy number, somatic mutations, and protein levels, circus plots were drawn to display the interrelation between *CHN1* and other genes in GC (Figure 5(a)). GSEA was applied to perform a hallmark analysis for *CHN1. CHN1* was found to be enriched under the terms ‘Focal adhesion’, ‘Extracellular matrix organization’, ‘Degradation of the extracellular matrix’, ‘ECM receptor’, and ‘PI3K-AKT signaling pathway’. Importantly, these pathways were remarkably upregulation in high-risk cases (Figure 5(b)). The expression of six epithelial–mesenchymal transition (EMT)-related factors – *TGFβ1, MMP2, MMP9, CDH1, CDH2*, and *VIM* – was found to be positively correlated to that of *CHN1* (P < 0.001) (Figure 5(c)).

### Tumor-infiltrating immune cells associated with CHN1 expression in GC

**Figure 6.**
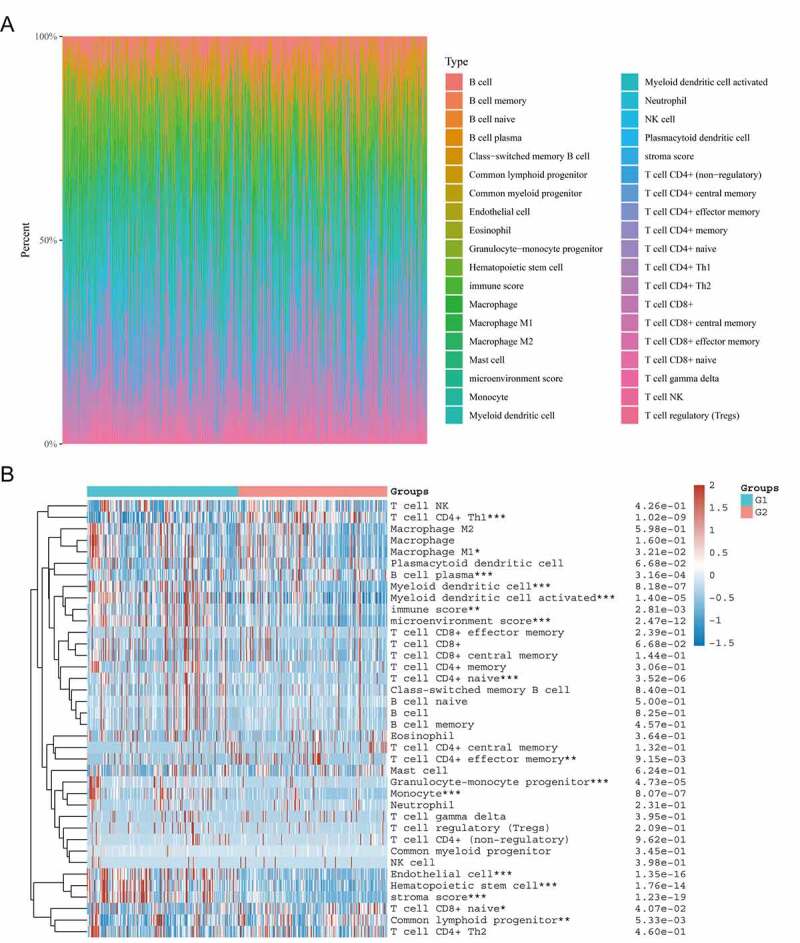
Correlation between *CHN1* expression and the infiltration of immune cells. (a) Immune cell score heat map, where different colors represent the expression trend in different samples. *P < 0.05, **P < 0.01, and ***P < 0.001, and the asterisk represents the degree of importance (*P). The significance in the two groups of samples passed the Wilcox test. (b) Percentage abundance of tumor-infiltrating immune cells in each sample, with different colors and different types of immune cells. The abscissa represents the sample, and the ordinate represents the percentage of immune in a single sample

As shown in Figure 6(a), the composition of 35 immune cell types varied significantly across samples. The CIBERSORT algorithm was used to evaluate the correlation between *CHN1* expression in the tumor microenvironment and tumor-infiltrating immune cells. We observed that the expression level of *CHN1* was correlated with the abundance of infiltration by CD4 + T cells, Th1 cells, M1 macrophages, plasma B cells, myeloid dendritic cells, activated myeloid dendritic cells, naïve CD4 + T cells, CD4+ effector memory T cells, granulocyte–monocyte progenitors, monocytes, endothelial cells, hematopoietic stem cells, naïve CD8 + T cells, and common lymphoid progenitors (P < 0.05). It was also positively correlated with the immune score, microenvironment score, and stroma score (P < 0.05) (Figure 6(b)).

### Significance of CHN1 in immunotherapy against GC

**Figure 7.**
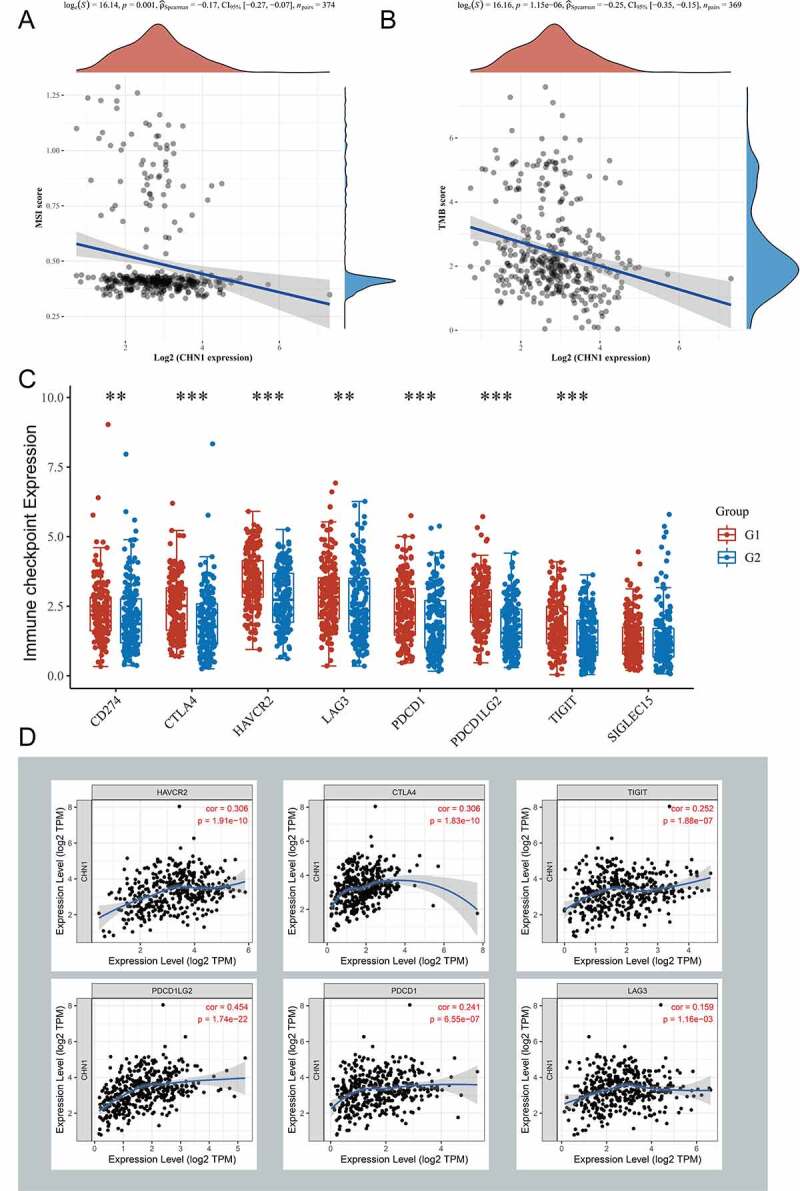
Significance of *CHN1* in immunotherapy against gastric cancer. (a) Correlation of *CHN1* expression with tumor mutation burden (TMB). (b) Correlation between *CHN1* expression and microsatellite instability (MSI). (c) Relationship between the expression of *CHN1* and that of common immune checkpoint proteins (ICPs). Red and ue represent the high and low *CHN1* expression groups, respectively (*P < 0.05, **P < 0.01, ***P < 0.001). (d) Spearman correlation coefficients for the relationship between *CHN1* expression levels and the expression levels of the six ICPs

At first, we calculated the correlation between *CHN1* levels and tumor mutation burden (TMB) as well as microsatellite instability (MSI) in GC and found that *CHN1* expression was negatively correlated with both (Figure 7(a) and (b)). In addition, we divided patients with GC into high- and low-*CHN1* expression groups, and found that *CD274, CTLA4, HAVCR2, LAG3, PDCD1, PDCD1LG2*, and *TIGIT* were differentially expressed in the high/low-*CHN1* expression group (Figure 7(c)). Finally, we calculated the correlation between *CHN1* expression and that of these immune checkpoint, and the results are shown in Figure 7(d).

### CHN1 methylation in GC

**Figure 8.**
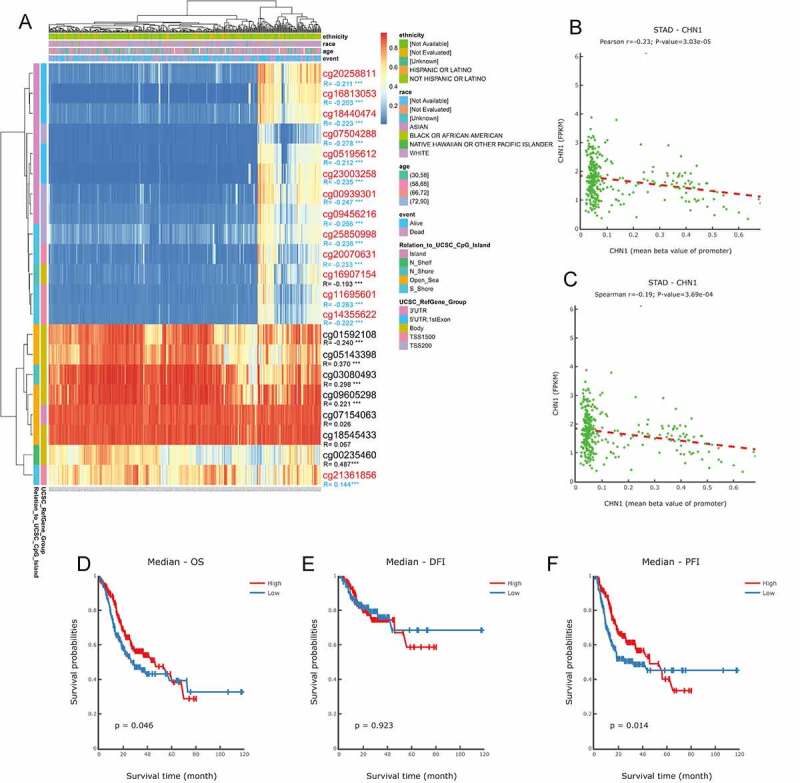
Analysis of *CHN1* methylation in gastric cancer (GC). (a)Waterfall plot for methylation of the *CHN1* gene. The correlations between *CHN1* methylation and expression were also analyzed. The red cg probe was located in the promoter region.(b-c) Pearson’s correlation coefficient (b) and Spearman correlation coefficient (c) for the relationship between the methylation level at the *CHN1* promoter and *CHN1* expression.(d-f) Overall survival (d), disease-free survival (e), and progression-free survival (f) based on methylation at the *CHN1* gene promoter; P < 0.05 was considered statistically significant

**Figure 9.**
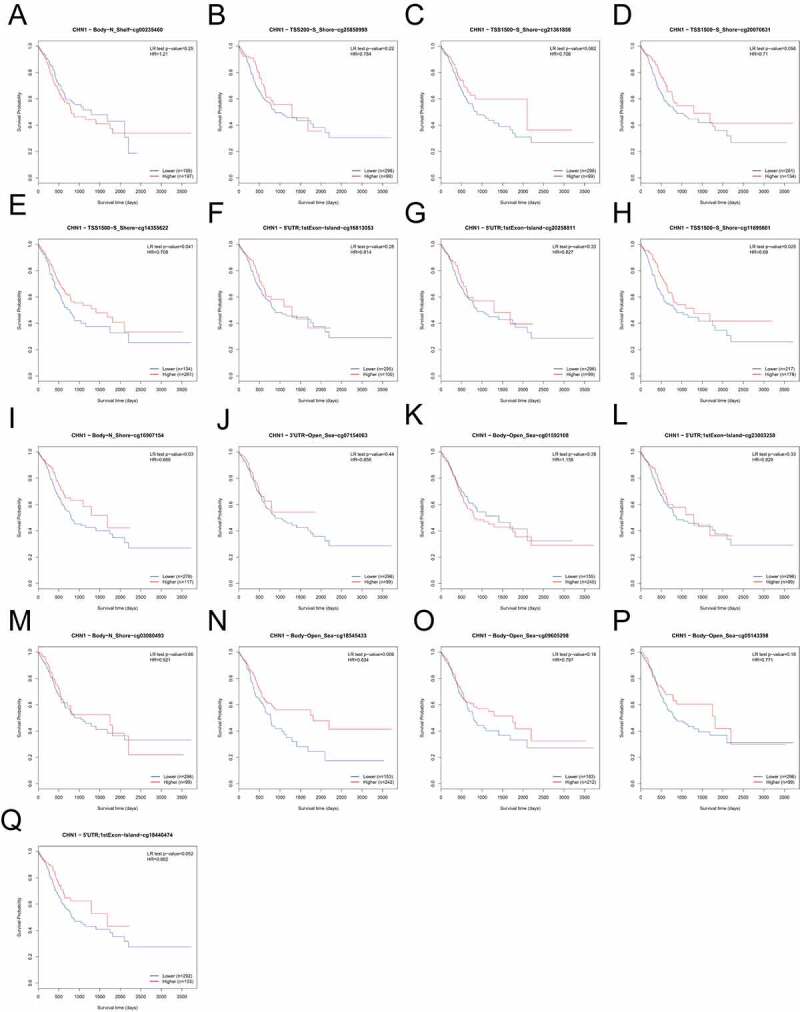
Survival analysis for all methylation probes; P < 0.05 was considered statistically significant

On the basis of methylation data from TCGA-STAD, we observed that methylation values from the methylation probes, cg20258811, cg16813053, cg18440474, cg07504288, cg05195612, cg23003258, cg00939301, cg09456216, cg25850998, cg20070631, cg16907154, cg11695601, cg14355622, and cg01592108 were negatively correlated with *CHN1* expression level, while those from methylation probes cg05143398, cg03080493, cg09605298, cg07154063, cg00235460, and cg21361856 were positively correlated with *CHN1* expression (Figure 8(a), p < 0. 05). The correlation between the degree of *CHN1* promoter methylation and *CHN1* expression levels revealed a negative relationship between the two (Figure 8(b-c)). Further, we observed that low levels of promoter region methylation were predictive of a poor prognosis (Figure 8(d-f)). Finally, when all methylation probes were included in the survival analysis, hypomethylation at cg14355622, cg11695601, cg16907154, and cg18545433 showed a statistically significant relationship with survival (Figure 9).

## Discussion

GC has had one of the highest mortality and morbidity rates among all malignant tumors over the last five decades [1]. GC development is a gradual process involving several factors such as genetic predisposition, dietary incontinence, *H. pylori* infection, smoking, alcohol consumption, and environmental pollution [28]. Not only does carcinogenesis in this disease occur via a complex array of genetic structural alterations and expression abnormalities, but these genetic changes are also interrelated and comprise a delicate biological network [29]. The genetic alterations in this network are not simply superimposed; cellular malignancies are triggered by abnormal functioning of a series of network systems regulating gene clusters that play a role in cell growth and differentiation and other pathways [30,31]. Fluctuations in any node can affect the equilibrium of the entire network. Therefore, we preliminarily explored how *CHN1* is involved in the development of GC based on bioinformatics.

To date, there have been few reports on *CHN1* in tumors, but *CHN1* has been found to play an oncogenic role in cervical cancer. Liu et al. showed that CHN1 may induce EMT through the Akt/GSK-3β/Snail signaling pathway, thus participating in the occurrence and development of cervical cancer [32]. In the present study, using public databases and IHC analysis, we demonstrated that the expression of *CHN1* was significantly higher in GC than in normal tissue. GC patients with a high expression of *CHN1* had a shorter survival duration than those with low expression. Taken together, these results indicated that *CHN1* may be an oncogene in GC too and may play an important role in the occurrence and development of GC. Additionally, *CHN1* expression levels differed between tumors at various T stages and could be used to differentiate T1 tumors from tumors at other stages. Accordingly, we constructed a PPI network centered around *CHN1* as the core gene and found an association between the expression of *CHN1* and *ERBB2*, which is involved in the pathogenesis and adverse consequences of various cancers, including terminal GC and gastro-esophageal junction cancer [33,34]. We then screened for genes co-expressed with *CHN1* using TCGA-STAD, and enrichment analysis suggested that *CHN1* and these genes may be involved in multiple pathways related to the extracellular matrix. Studies have demonstrated that CHN1 shows synergistic effects with Rac1 and Cdc42 Hs, which can induce the formation of lamellipodia and filopodia. This is the foundation of cell migration, and it is also a key step in tumor cell migration and invasion [35,36]. EMT, which is achieved through a disruption of cell-to-cell adhesion, regulation of cell-matrix interactions, cytoskeletal rearrangement, and acquisition of migration capacity [37], is also related to the transformation of early noninvasive tumors into aggressive malignant tumors [38]. Further, our co-expression analysis also showed that key EMT-related factors such as *MMP2, MMP9, CDH2, TGFB1*, and *VIM* were highly expressed when *CHN1* was highly expressed, while *CDH1* was expressed at lower levels, suggestive of a potential cancer-promoting function of *CHN1*.

Immunotherapy is currently a hot topic in tumor research [39,40]. Our study showed that *CHN1* expression had a significant relationship with infiltration by CD4 + T cells, M1 macrophages, monocytes, plasma B cells, endothelial cells, dendritic cells, and hematopoietic stem cells. We further explored the correlation between *CHN1* expression and common immune checkpoint and found positive correlations between the expressions of *CHN1* and *CD274, CTLA4, HAVCR2, LAG3, PDCD1, PDCD1LG2*, and *TIGIT*. In addition, we also found that *CHN1* expression was negatively correlated with TMB and MSI. Multiple studies have analyzed in detail and demonstrated the high correlation of TMB and MSI with immunotherapeutic efficacy [41,42]. Together, these results suggested that *CHN1* expression may affect the effectiveness of immunotherapy

DNA methylation is an important epigenetic modification that is closely related to tumor occurrence and development [43–45]. It is well-known that hypermethylation within the promoter region leads to the inactivation of some tumor-suppressor genes. Many studies on different cancer types have shown that a large number of genes are silenced by DNA methylation [46]. Therefore, we evaluated the levels of *CHN1* DNA methylation in GC. We found that the degree of methylation at the *CHN1* promoter was negatively correlated with *CHN1* expression and that hypomethylation of the promoter region was predictive of a poor prognosis in GC patients. Further, we also found that hypomethylation at cg14355622, cg11695601, cg16907154, and cg18545433 was correlated with a poor prognosis in GC patients. Further studies are warranted to elucidate how methylation at different sites in the *CHN1* gene affects its expression and survival outcomes in GC patients.

Taken together, our results demonstrate for the first time that *CHN1* encourages GC development by controlling several signaling pathways, and its expression is correlated with immune cell infiltration. However, there are some shortcomings and limitations to our study. First, our study was based on information from public databases and published literature, and uneven data quality may have affected our results. Second, the differences in accuracy among the databases used for data analysis and the choice of statistical methods may affect the interpretation of our research results. Nevertheless, we verified our research by analyzing multiple databases and data sets, datasets and obtained similar results, which points to the validity of our study.

## Conclusion

Our study confirmed that the levels of *CHN1* were higher in GC cells and tissues than in normal gastric epithelial cells and tissues. A high expression of *CHN1* was associated with a poor prognosis and with some clinicopathological features of GC. Multivariate analysis revealed that upregulated *CHN1* expression in GC is an independent risk factor for shortened OS. Our results showed that high *CHN1* expression may regulate GC progression of gastric cancer through via multiple pathways and that *CHN1* levels may influence the effectiveness of immunotherapy in GC. Our results suggest that the expression of *CHN1* may be a diagnostic and prognostic indicator in GC and could be a potential target for GC treatment.

## Data Availability

We promise that all the data in this article are authentic, valid and available.
